# Effect of Physical Activity Interventions on Health Parameters in Children and Adolescents with Intellectual Disabilities: A Systematic Review

**DOI:** 10.3390/healthcare12232434

**Published:** 2024-12-03

**Authors:** Luis Maicas-Pérez, Juan Hernández-Lougedo, José Luis Maté-Muñoz, Ariel Villagra-Astudillo, Pablo García-Fernández, Borja Suárez-Villadat, Blanca Jiménez-Rojo

**Affiliations:** 1 NÌKE Research Group, Faculty of Health Sciences, International University of La Rioja, 26006 Logroño, Spain; lmaicas@atleticodemadrid.com (L.M.-P.); jmate03@ucm.es (J.L.M.-M.); ariel.villagra@uam.es (A.V.-A.); pablga25@ucm.es (P.G.-F.); bsuarvil@uax.es (B.S.-V.); blancajimenezrojo@gmail.com (B.J.-R.); 2 Innovation and Health Department, Club Atlético de Madrid Foundation, 28022 Madrid, Spain; 3 Physiotherapy and Health Research Group (FYSA), Faculty of Health Sciences—HM Hospitals, University Camilo José Cela, 28692 Madrid, Spain; 4 Instituto de Investigación Sanitaria—HM Hospitales, 28692 Madrid, Spain; 5 Faculty of Nursing, Physiotherapy and Podiatry, Complutense University of Madrid, 28040 Madrid, Spain; 6 Department of Physical Education, Sport and Human Motricity, Autónoma University of Madrid, 28049 Madrid, Spain; 7 Department of Physical Activity and Sports Sciences, Alfonso X el Sabio University, 28691 Madrid, Spain

**Keywords:** intellectual disability, psychomotor development, adapted sports, inclusive environment, adapted physical activity, health, quality of life impact, social inclusion, systematic review

## Abstract

Intellectual disability (ID) encompasses diverse challenges that affect daily life and health. Sedentary behaviors, prevalent in this population, contribute to alarming health concerns, notably obesity and musculoskeletal issues. This review examines the role of physical activity (PA) interventions in addressing these health challenges among children and adolescents with ID. This systematic review followed the Preferred Reporting Items for Systematic Reviews and Meta-Analyses (PRISMA) guidelines. The search was conducted in the Cochrane Library, PubMed, Scopus, and SPORTDiscus databases, using specific keywords aligned with the PICO framework (population, intervention, comparison, and outcome). From January 2013 to October 2024, a total of 5236 studies were identified, of which 17 met the inclusion criteria for this review according to PRISMA procedures. Randomized controlled trials (RCTs) focusing on the impact of PA on body composition, physical fitness, bone health, metabolic indicators, and overall quality of life were included. The study cohort consisted of individuals aged 13–24 diagnosed with ID. The findings consistently highlight the positive relationship between PA interventions and improved health markers in individuals with ID. Diverse PA interventions, ranging from strength training to high-intensity exercises, demonstrated significant improvements in body composition, physical fitness, and bone mineral density. Notably, higher-frequency PA programs (minimum three sessions per week) yielded more substantial benefits. This review underscores the potential of adapted PA interventions to address health concerns and enhance the quality of life for individuals with ID. Further comprehensive research is needed to establish standardized guidelines for effective PA interventions in this population.

## 1. Introduction

Intellectual disability (ID) is characterized by a neurological dysfunction that represents a limitation in the development of activities and/or participation [1]. The etiological factors of ID are diverse and can be classified as genetic, acquired (either congenital or developmental), environmental, and sociocultural [2,3]. The phenotypic and genetic heterogeneity of this disability translates into 1700 pathologies considered as such [4].

**Figure 1:**
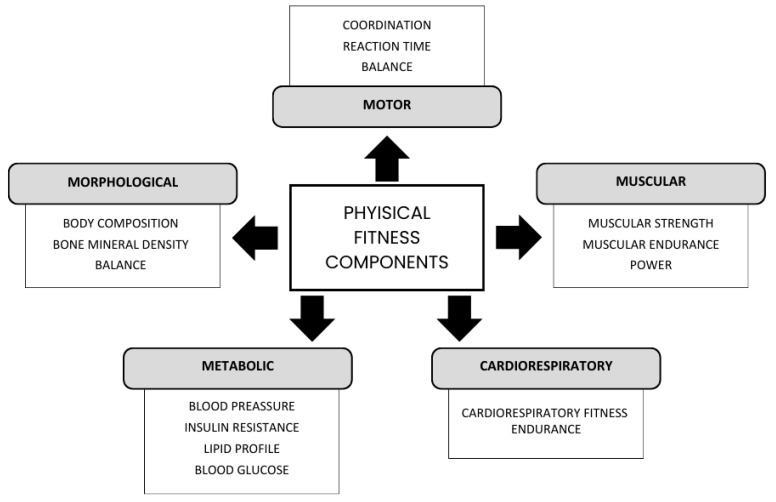
Components and factors of PA. Authors’ own elaboration. Note: Components that determine a person’s FC, developed by Bouchard 1994 [9].

To determine the health status of an individual, we cannot only focus on the absence of disease or pathology; health implies complete physical, mental, and social well-being [5]. The benefits of PA are demonstrated not only in metabolic components and/or physical health but also in the mental health and social life of individuals [6,7]. From the physical health point of view, physical fitness (PF) helps us to determine the extent of an individual’s health [8], assessing metabolic, morphological, locomotor, muscular, and cardio-respiratory components [9]. As a result, the measurement of PF has been greatly simplified, developing different tests (Figure 1).

Taking into account that the population with ID represents 2% of the global population [10], that their life expectancy has increased in recent years [11], and that the health problems they face with age are similar to those of people without ID [11], it seems interesting to bring the benefits of sport closer to children and young people, to ensure that they are likely to maintain a good quality of life as they age [12]. The WHO 2020 recommendations for this group are shown in Table 1 [13].

Despite the above, no systematic review has previously compared the effects of different types of PA on a variety of health parameters in children and adolescents with ID. Consequently, this review aims to answer the following research questions:

- What types of exercises have been being used as PA in children and adolescents with ID?
- What health parameters have been used as outcome indicators to determine the quality of life of these subjects?
- How does PA contribute to the improvement of health and quality of life in children and adolescents with ID?

The main objective of this systematic review is to determine which sports modalities have been used to date in the scientific literature as tools to promote the health and quality of life in children and adolescents with ID; and, in addition, to verify which variables have been used to determine the changes that have occurred in them.

## 2. Materials and Methods

The development of the systematic review followed the guidelines of the Preferred Reporting Items for Systematic Reviews and Meta-Analyses (PRISMA) [14]; PRISMA Checklist is available in Supplementary Materials. The review protocol is registered under the number CRD42023488366 in the PROSPERO database. No amendments to the original registration were made.

### 2.1. Search Strategy

A systematic search of randomized controlled trials (RCTs) that included any physical activity intervention in people with ID which contributed to health was performed. Four databases were consulted: Cochrane Library, PubMed, Scopus, and SPORTDiscus (last update: October 2024).

The search responded to the PICO question (population, intervention, comparison, and outcome): ‘What is the impact of the implementation of PA and/or sport protocols on the health and quality of life among children and adolescents with ID?’.

Thus, the search statement was developed as follows: (‘intellectual disability’ OR ‘Down syndrome’ OR ‘autism’) AND (‘physical activity’ OR ‘exercise’ OR ‘sport’) AND (‘health’ or ‘quality of life’).

### 2.2. Eligibility Criteria and Study Selection

To be included in the analysis, the studies had to meet the following criteria: (1) population: people (humans) with ID between 13 and 24 years old; (2) intervention: must perform any type of PA or sport; (3) outcome: variables regarding health or quality of life needed to be measured; and (4) year of publication: only articles published between the years 2013 and 2024 were included; books and doctoral theses are also valued.

Exclusion criteria encompassed non-peer-reviewed studies, studies without a control group, and studies lacking quantitative outcome measures. Additionally, studies that focused on evaluating the impact of interventions on family members, therapists, or other stakeholders—rather than on the individuals with disabilities themselves—were excluded. Articles describing interventions that were not implemented, such as theoretical proposals or study designs without data, were not considered either. No language restrictions were applied, although all included studies were in English. Books, chapters, and doctoral theses were also reviewed during the search, but none met the eligibility criteria.

After conducting the search in the databases, the results of these four were grouped and the duplicated articles were eliminated. The titles of the articles were reviewed and those whose titles were not related to the subject of the question were discarded. The abstracts of the remaining articles were then analyzed. Finally, a total of 57 complete articles were reviewed. Two authors reviewed the studies for inclusion, and discrepancies and questions were forwarded to a third author for consultation, who finally decided on the definitive inclusion of one or the other article.

### 2.3. Data Extraction

To make the review and analysis possible, different parameters were extracted from the text.

One of the investigators was responsible for reading, analyzing, and extracting the information from the articles based on a pre-drafted list of items that included the characteristics of the participants and whether they were distributed in groups, the exercise program, the outcomes and measurement tools, and the results or main findings of the studies.

Subsequently, three investigators reviewed and confirmed the accuracy of the extracted data.

## 3. Results

### 3.1. Included Studies

After reviewing the databases, a total of 5236 articles and 4 books and doctoral theses were identified, of which 2420 remained after excluding duplicates. After applying the exclusion criteria, 57 articles were obtained, of which only 17 were selected for inclusion as they successfully met the inclusion criteria. These criteria were, in addition to those mentioned above, that the protocols had already been implemented and that they were targeted at and assessed individuals with disabilities.

**Figure 2:**
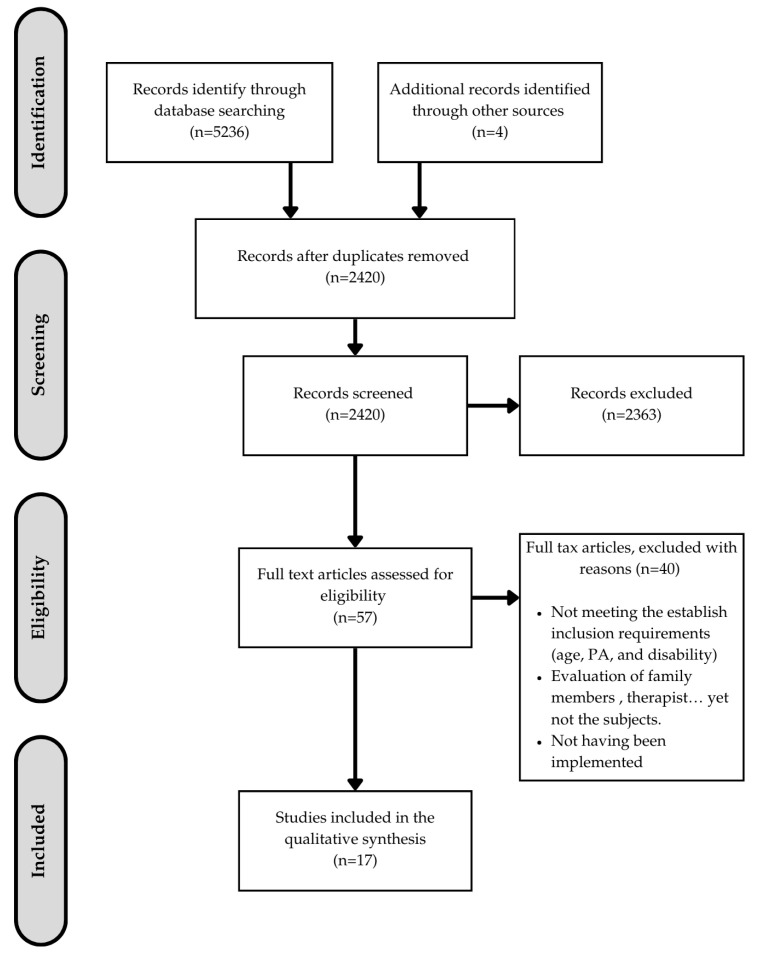
Flow chart of the systematic review process.

This process of identification, screening, selection, and inclusion is shown in Figure 2 and specifically analyzed in Appendix A.

### 3.2. Characteristics of the Studies

#### 3.2.1. Types of Studies

All the studies included in this systematic review share the following common characteristics: they respond to the PICO question posed, as well as meeting the inclusion criteria.

A total of 14 RCTs, with implementation times ranging from 8 weeks to 12 months, were included. Within these studies, the following evaluations were conducted: body composition (8 articles), physical condition (8), PA levels (4), metabolic condition (4), BMD (3), diet (2), and neuropsychological and psychomotor capacity (1).

#### 3.2.2. Sample

The participants of the studies met the conditions established in the PICO question. The population consisted of people (humans) with ID between 13 and 24 years of age.

The largest sample within the studies included in the review was from [15] with 110 subjects, and the smallest was from [16] with 21. Out of all the studies, eight of them included unspecified ID and nine Down syndrome (DS), resulting in 502 subjects with ID and 335 with DS.

#### 3.2.3. Measuring Instruments

Morphological components:

Body composition: eight of the studies evaluated the body composition of the subjects.

The variables assessed were height, weight, BMI (10), waist circumference (7), waist-to-height ratio (3), fat mass (8), fat-free mass (1), and skinfold sum (3).

Instruments: electrical bioimpedance (4), DXA (1), plicometer (3), and formulas (10).

Bone health: three of the studies assessed this condition based on BMD and bone mineral content (BMC).

For this purpose, all three studies used dual X-ray absorptiometry (DXA), and one of them complemented it with broadband ultrasound attenuation (BUA), quantitative bone ultrasound (QUS), and ultrasound conduction velocity (SOS).

Muscular and cardiorespiratory components:

Eight were studies that assessed physical and cardiorespiratory components (1, 2, 4, 5, 7, 8, 10, and 11).

The variables measured were aerobic capacity (7), strength (10), flexibility (5), speed (4), stability (2), power (1), and cadence (1).

For this, they used the following instruments: spirometry (2), tests assessing PF (6′ walk test, 30″ sit to stand, sit and reach, etc.) (11), dynamometry (8), and computer cycle (adapted bicycle) (1).

Metabolic components:

These were assessed in two of the studies.

The components were blood pressure (1), lipid profile (1), HOMA-IR insulin resistance index (1), systolic blood pressure (SBP) (1), and diastolic blood pressure (DBP) (1).

Instruments: blood test (1), HOMA-IR (1), and manual sphygmomanometer (1).

PA levels and diet:

PA levels: in five of the studies the daily PA levels of the subjects were assessed. The variables used were heart rate (HR) variability, minutes per day, and qualitative variables based on questionnaires.

The measurement instruments used were questionnaires (2), accelerometer (2), and HR monitor (1).

Diet: dietary habits were evaluated in two studies. Nevertheless, four of them included a diet in their implementation protocol.

The variables included food intake.

The instruments used were a 7-day record questionnaire and a 3-day one. Neuropsychological and psychomotor capacity: a single study evaluated this capacity.

The variables determined were the number of hits based on time and manual dexterity after a cycling intervention.

The tests used were the Purdue Pegboard Test (PPT) and the Tower of London Test (ToL).

#### 3.2.4. Physical Activity Protocols Used

The included studies reported a wide range of intervention characteristics, providing valuable insights into the structure and implementation of PA programs for individuals with ID.

Intensity:

The intensity of the interventions was reported in 12 out of the 17 included studies. It was commonly measured using heart rate (e.g., the percentage of maximum heart rate or heart rate reserve), perceived exertion scales, or specific training protocols involving progressive resistance. For example, seven studies used heart rate measures to quantify intensity, and five studies incorporated progressive resistance protocols. Aerobic activities were generally performed at moderate intensity (40–70% heart rate reserve), while strength-training programs utilized progressive increases in resistance or workload.

Duration:

The duration of individual training sessions was reported in 15 studies, ranging from 20 to 90 min. Among these, 11 studies implemented sessions lasting between 40 and 60 min, which typically included a structured warm-up, a core exercise phase, and a cool-down period.

Frequency:

Weekly session frequency was reported in 16 studies. Regarding frequency, two and three sessions per week predominated (with seven studies each) and, to a lesser extent, five or more days of PA (two studies). In the latter cases, the participants were asked to do moderate activity, without specifying the type of PA.

Types of Exercise:

The types of exercise interventions were described in all 17 studies. Aerobic activities, such as cycling, swimming, and walking, were utilized in 10 studies, while strength-based programs involving resistance machines, free weights, or bodyweight exercises were implemented in 8 studies. Additionally, five studies employed mixed programs that combined aerobic and strength components, and two studies used innovative approaches such as exergaming. Whole-body vibration (WBV) was also featured in two studies to target specific outcomes, such as improving bone health. This diversity highlights the adaptability of PA programs to meet individual needs and preferences within this population.

### 3.3. Risk of Bias: Quality of the Studies

Table 2 shows the quality assessment of the studies that were finally included in this systematic review. The risk of bias in each of the RCTs was determined using the PEDro scale in all articles; one of the studies obtained acceptable quality (Boer), while the rest were rated as good.

All studies (100%) fulfilled items 2, 4, and 11 (2. Random allocation; 4. Baseline comparability; 11. Reporting of point measures and measures of variability). In contrast, none of the studies fit criteria 5 and 6 (5. Blinding of participants; 6. Blinding of therapists), and only three met criterion 9 (intention-to-treat analysis). Item 10 (between-group statistical comparisons) was met by 94%, followed by item 1 (eligibility criteria) with 82% and item 8 (adequate follow-up (>85%) met by thirteen studies or 76%. Finally, criteria 3 and 7 (3. Concealed allocation; 7. Blinding of assessors) were met by 6 and 10 of the studies, respectively, which equals to 35% and 59%. Adequate follow-up is particularly important for minimizing attrition bias, while concealed allocation helps prevent selection bias.

In conclusion, while the studies generally followed rigorous randomization and statistical analysis procedures, the lack of blinding and intention-to-treat analysis are important limitations that must be considered in the development of the systematic review. After analyzing the articles included in this review, it can be seen that we found items 5, 6 and 7 in the evaluation of the PEDro scale to be a limitation, explicitly recognizing this as an important limitation in the development of the systematic review carried out.

Although publication bias is a critical consideration in systematic reviews, a funnel plot analysis was not conducted as the number of included studies (*n* = 17) was insufficient for a meaningful evaluation.

## 4. Discussion

This systematic review focused on analyzing the role of PA and exercise in the health of children and adolescents with ID, from a comprehensive point of view. The information provided by the 17 studies included in this review, their effects, and implications were evaluated.

These studies provide consistent evidence that PA can have a positive impact on various components of health in people with ID, such as improved body composition, PF, and BMD. These results support the idea that PA promotion can play a crucial role in health care in this population group [32].

Body composition is one of the most assessed variables to date in this group [30,33]. The measurement tools for this variable include electrical bioimpedance, DEXA, and BMI. It is important to highlight, however, that BMI does not always reflect body fat stores/deposits; it includes both fat mass (FM) and lean mass (LM), and, in some cases, the increase in this value may be due to muscle development and increased LM, rather than an accumulation of adipose tissue stores in the body [34]. Only two of the eight articles evaluating this component used BMI exclusively. One of them focused on the assessment of neuropsychological and psychomotor capacity, limiting adiposity to a secondary variable. PA could be useful for the reduction in %BF, as well as for the improvement of body composition [35].

In this regard, it seems more interesting to apply high-intensity workouts as opposed to light- or moderate-intensity ones. Similarly, 3 days a week may have more noticeable improvements than 2 days a week. This has been previously proved, showing that a duration of over 8 weeks of high-intensity intervallic training is more effective in reducing fat mass and fat-free mass (FM and FFM) than a shorter duration, indicating the need for a prolonged stimulus to reduce BF and promote muscle mass remodeling, as well as three weekly sessions being more effective on body composition than two weekly sessions [35].

Furthermore, recent studies highlight the value of different PA modalities in improving health markers in adolescents with DS. It has been shown that a 20-week exergame program, administered three times per week, led to significant improvements in health-related fitness and body composition in adolescents with DS [26]. This innovative approach suggests that interactive, game-based PA, such as exergames, could offer a highly engaging and effective intervention for this population, particularly in improving adherence and motivation.

Another study compared the effects of aerobic versus strength training, demonstrating that strength training led to significant improvements in health-related fitness but not body composition [27]. This reinforces the idea that different PA types yield varied results: while strength training is particularly effective for improving physical fitness, it may not be as impactful for body composition in DS adolescents. This evidence suggests that strength-based programs can be prioritized for physical fitness gains, while other interventions may be necessary to address body composition outcomes.

The most recent study on a 16-week swimming program emphasized swimming as a unique and highly beneficial activity for adolescents with DS [28]. Compared to strength training and a combined swim–strength approach, swimming alone resulted in significant improvements in both fitness and body composition. This may be due to the unique cardiovascular and muscular demands of swimming, which could offer an optimal balance of aerobic and anaerobic benefits for DS adolescents. Given that swimming is also a popular and accessible activity, these findings support its potential for widespread implementation in PA programs for this population. Furthermore, 8 weeks seems to be sufficient time to improve strength (1 RM) in both the upper and lower body in this population. This has been demonstrated in other research, in which two weekly sessions focused on strength work resulted in significant muscle improvements [36].

Combined training and high-intensity training have also been shown to be useful for the improvement of maximal oxygen consumption and thus cardiorespiratory fitness [37,38,39]. PA can improve the physical capacity of people with ID.

Bone health, an essential aspect of our overall well-being, plays a crucial role in our mobility and quality of life; the mineralization of our bones peaks in youth; peak bone mass is around age 20 [40]. To date, exercises that concern gravitational and impact forces, such as osteogenic ones, are crucial in the acquisition of a higher BMD, which will lead to a decrease in the risk of suffering any bone pathology [40,41,42]

A prominent approach in scientific literature is whole-body vibration (WBV). This technique, which involves mechanical vibrations transmitted to the body, has shown promising results in improving bone health. However, the efficacy of WBV depends on several factors, such as vibration frequency, wavelength, and the duration of exposure [43]. The optimal frequency seems to be around 30 Hz [42]. In addition, efficiency in terms of time and benefit is an important aspect to consider, as WBV can provide improvements in bone health in a relatively short period [36]. Although gains may be more noticeable in individuals without DI, PA remains a valuable component of maintaining good bone health in all populations. Options such as WBV and osteogenic exercises can be effective, but it is essential to tailor approaches according to individual needs [44].

The inclusion of the assessment of neuropsychological and psychomotor ability is a salient feature in one of the articles. This reflects a more holistic understanding of the effects of PA in individuals with ID. However, it is an area that requires further attention and development to fully understand how PA can influence broader aspects of their well-being and quality of life.

It is also interesting to note the variety of approaches that have been taken in the implementation of programs involving PA, including strength training, aerobic exercise, combined exercise, and even cycling and swimming. These approaches demonstrate the versatility and feasibility of possible interventions. This diversity is valuable, as it allows us to adapt PA programs according to the individual preferences, needs, and characteristics of people with ID, which are highly diverse due to the heterogeneity of this group [45].

In this regard, it is relevant to stress that the studies also show certain limitations. The varied methodological quality and limitations in the experimental design in some cases can affect the accuracy of the results. This can be observed in studies in which PA was not delivered face-to-face, with a guide. The role of parents/people close to people with ID in their self-control or engagement has been evidenced in several studies, such as greater adherence to the diet if there is parental supervision [16], or greater performance and self-control if they have a person by their side in a sporting event [46]. It appears that an intervention in which participation is required and there is a guide has greater improvements than one in which only criteria/guidelines are requested to be met without direct supervision [16,46].

There are other elements that could have a significant impact on this population and have not been thoroughly analyzed. For example, adherence levels to the program seem to be a key element in this type of study. However, only a few studies report this information [23,25,47], such as those by [23,25] and Moraes et al. (2022) [47], which show adherence rates to sessions of 90%, 92%, and 100%, respectively. Another important factor could be the previous experience of participants in different studies. We only found the study by Moraes et al. [47], which states that participants had prior experience with commercial non-immersive virtual reality games.

Dropout rates have also been analyzed in this review. This variable has indeed been reported in most studies, being attributed to diverse causes such as injuries, educational commitments, or even transportation issues [16,17]. However, other aspects, such as gender differences or the use of medication, have not been addressed in the studies. A possible explanation for this might lie in the exclusion criteria of the studies, where participants with epilepsy or severe health problems are often excluded.

In conclusion, the lack of assessment of these variables underscores the need to conduct studies that accurately evaluate the effects of medication, adherence, and other factors on the health of individuals with ID.

According to the analysis of the different articles, guided physical activity is of vital importance for the improvement of quality of life, adherence to exercise, body composition, and health of people with ID, and the role of close people who accompany them in their daily lives is also relevant, so it would be interesting to update the guidelines on physical activity in this population group, making them reach associations, working groups, companies, and families that have a direct link with ID.

Issues of blinded allocation and assessment, as well as sample heterogeneity, could introduce biases and limit the accuracy of findings. It is essential that future research address these limitations and employ more robust approaches to assess the effects of PA in this population.

## 5. Conclusions

In conclusion, the results of this systematic review have consistently shown that PA plays an important role in the improvement of body composition, PF, and BMD, so that the implementation of such PA programs, particularly those focused on high intensity, produces significant impacts in the reduction in %BF and in the improvement of muscle strength and cardiorespiratory capacity, which are determining factors in the quality of life and health of people with ID.

It has been observed that PA programs with a frequency of at least three weekly sessions tend to be more effective in obtaining significant improvements in body composition and muscle strength, and, as has been indicated throughout this review, the role played by people and/or the environment surrounding people with ID is fundamental for the adherence and achievement of successful PA programs.

This is why PA should be implemented in groups, associations, companies, and other environments that include people with ID for the improvement of their daily life.

## Figures and Tables

**Table 1:** Recommendations of the World Health Organization, 2020 [13].

Children (5–17 Years Old)
At least an average of 60 min of daily moderate-to-vigorous-intensity PA (mainly aerobic) throughout the week.
Muscle- and bone-strengthening activities at least three days a week.

Note: This table shows the volume and intensity of PA recommended for people with ID by WHO.

**Table 2:** Assessment of the quality of the studies. PEDro Scale.

Article/Study		1	2	3	4	5	6	7	8	9	10	11	Total Score	Confirmed PEDro Database	Quality
Boer et al. [17]	2014	+	+	-	+	-	-	-	+	-	+	+	5	Yes	Acceptable
Curtin et al. [16]	2013	+	+	-	+	-	-	-	+	-	+	+	5	Yes	Acceptable
Ferry et al. [18]	2014	+	+	-	+	-	-	-	-	-	+	+	4	Yes	Acceptable
Holzapfel et al. [19]	2015	+	+	-	+	-	-	-	-	-	+	+	4	Yes	Acceptable
Lee et al. [20]	2016	+	+	-	+	-	-	+	+	+	-	+	6	Yes	Good
Matute-Llorente et al. [21]	2015	+	+	-	+	-	-	-	-	-	+	+	4	Yes	Acceptable
Matute-Llorente et at. [22]	2015	+	+	-	+	-	-	-	-	+	+	+	5	Yes	Acceptable
Naczk et al. [23]	2021	+	+	-	+	-	-	-	+	-	+	+	5	Yes	Acceptable
Ptomey et al. [24]	2021	+	+	+	+	-	-	+	+	-	+	+	7	Not available	Good
Ptomey et al. [15]	2022	+	+	+	+	-	-	+	+	-	+	+	7	Not available	Good
Shields et al. [25]	2013	+	+	+	+	-	-	+	+	+	+	+	8	Yes	Good
Suarez-Villadat et al. [26]	2023	+	+	+	+	-	-	+	+	-	+	+	7	Yes	Good
Suarez-Villadat et al. [27]	2024	+	+	+	+	-	-	+	+	-	+	+	7	Not available	Good
Suarez-Villadat et al. [28]	2024	+	+	+	+	-	-	+	+	-	+	+	7	Not available	Good
Sun et al. [29]	2022	+	+	-	+	-	-	+	+	-	+	+	6	Yes	Good
Wang et al. [30]	2022	+	+	-	+	-	-	+	+	-	+	+	6	Yes	Good
Yu et al. [31]	2022	+	+	-	+	-	-	+	+	-	+	+	6	Yes	Good

Note: 1. Eligibility criteria and source (this item does not contribute to total score); 2. Random allocation; 3. Concealed allocation; 4. Baseline comparability; 5. Blinding of participants; 6. Blinding of therapists; 7. Blinding of assessors; 8. Adequate follow-up (>85%); 9. Intention-to-treat analysis; 10. Between-group statistical comparisons; 11. Reporting of point measures and measures of variability. (Studies marked as “Not available” were manually evaluated due to the lack of data in the PEDro database for those studies. Studies marked as “Yes” were evaluated directly using the PEDro database, and the scores were verified.) (“+” indicates that the criteria has been met, whereas “-” the opposite).

## Data Availability

We did not create new data.

## Supplementary Materials

The following supporting information can be downloaded at: https://www.mdpi.com/article/10.3390/healthcare12232434/s1, PRISMA Checklist.

## Appendix A

**Table A1:** Articles added in the systematic review. “*” means that there are significatnt improvements in BMC; and “†” means that these significant improvemnts is related to the BMD.

Author	Year	Population	Protocol	Outcome	Results
Boer et al. [17]	2014	**Disability**: ID **Age**: 17 (±3) **Sample**: 54 - **Sprint Interval Training (SIT):** 17 - **Circuit Aerobic Training Continuo (CAT):** 15 - **Control Group (CON):** 14	**Length:** 15 weeks **Activity:** cycling, walking/running, and stepping **Volume:** 40′/session (5′ warm-up, 30′ work, 5′ cool-down) **Frequency:** 2 days/week **Intensity:** - **SIT:** 3 blocks of 10 min at ventilatory threshold (VT) (Blocks 1 and 3: 10 sprints of 15 s, followed by 45 s of relative rest; **block 2:** continuous training). After 8 weeks, intensity was increased to 110% of VT (cycling). - **CAT:** 3 blocks of 10 min of continuous training (cycling (1); walking/running (2); step (3)). HR similar to VT. After 8 weeks, intensity was increased to 110% of VT.	**Body composition:** Height, weight, BMI, waist circumference, fat mass, and fat-free mass. **PF:** Exercise stress test (ramped protocol (+15 W/min) starting at 30 W) HR, ECG, blood pressure, respiratory gas measurements with a Metalyzer 3B (Cortex, Leipzig, Germany). Oxygen consumption (VO_2_), carbon dioxide production (VCO_2_), minute ventilation, tidal volume, respiratory rate, and mixed expiratory CO_2_ concentration. Lower extremity strength (sit-to-stand). 6 min walk test (6 mW). Muscle fatigue endurance (hand dynamometer). **Metabolic fitness:** Blood pressure, lipid profile, and HOMA-IR insulin resistance index.	Significant improvements (POST) in: **CAT vs. CON:** **Body Composition:** % fat and waist circumference *. **Lipid profile:** HOMA-IR *. **Physical condition:** VT (W) *, VT (VO_2_) *, 6 mW *, and resistance to muscle fatigue *. **SIT (vs. CON):** **Body Composition:** % fat * and waist circumference *. **Blood pressure:** SBP *. **Lipid profile:** Cholesterol *, HDL *, LDL *, Tri *, Insulin *, and HOMA-IR * **Physical condition:** VO_2_ max *, peak power (W) *, VT (W) *, VT (VO_2_) *, 6 mW *, and fatigue endurance *. **SIT vs. CON:** **Body Composition:** % fat *. **Blood pressure:** SBP *. **Lipid profile:** LDL * and Insulin *. **Physical condition:** VO_2_ max *, peak power (W) *, VT (W) *, and VT (VO_2_) *
Curtin et al. [16]	2013	**Disability:** Down Syndrome **Age:** 13–26 **Sample:** 21 - **Formative intervention in nutrition and PA (n = 10) (FORM)** - **Formative intervention in nutrition and PA + behavioral intervention (n = 11) (FORM+ INT)**	**Length:** 6 months (intervention) + 6 months (after the implementation) **Activity:** formative **Volume:** not specified **Frequency:** not specified **Intensity:** not specified	Evaluation at 10 weeks, 6 months, and 1 year. **Body composition:** sBody weight Body fat % (electrical bioimpedance) **Diet:** Intake of fruits, vegetables, and snacks with high energy and low nutrient content, by a 3-day food record questionnaire. **PA levels:** Moderate/vigorous physical activity (accelerometer).	**Body composition:** **Weight (kg):** At 6 months FORM + INT vs. FORM * (*p* = 0.005). Mean differences between groups were maintained at one year (*p* = 0.002). **%Fat:** There were no significant differences. PA level: At 6 months *, it increased in the FORM + INT group (*p* = 0.01) and decreased in the FORM group (*p* = 0.30). **Fruit intake:** There were no significant differences. **Vegetable intake:** At one year, FORM + INT vs. FORM * (*p* = 0.009). **Snack intake:** No significant differences.
Ferry et al. [18]	2014	**Disability:** Down syndrome - **Age:** (16.9 ± 1.5) (16 ± 1.8) **Sample:** 42 - **Control group: 22 (CON)** - **Training group: 20 (TRAIN)**	**Length:** 12 months **Activity:** osteogenic activities (plyometric jumps, weight training exercises, sprints, slalom, obstacles, and gymnastics routines, all in the form of dynamic games) **Volume:** 60 min/session (15′ warm-up + 40′ exercise + 5’ cool down) **Frequency:** 2 days/week **Intensity**: moderate to vigorous.	**Body composition:** Age, height, body weight, BMI, skinfold sum (cm), and %Fat. **Diet and level of physical activity:** 7-day record food questionnaire. **BMD and BMC:** Quantitative ultrasound bone scanning (QUS). Bone mass measurements were performed by dual X-ray absorptiometry (DXA) at the spine and hip, and ultrasound attenuation (BUA) and velocity (SOS) were assessed from the calcaneus using the QUS device. **PF:** EUROFIT (4 exercises). Length jump, sit and reach, sit-ups, and manual dynamometry.	At 12 months, significant differences: TG vs. CON. % body fat (0.02) BMD: lumbar spine (7%, *p* < 0.005) and total hip (10%, *p* < 0.05). BMC: lumbar spine (4%, *p* < 0.05). Trained individuals improved their physical fitness as assessed through the Eurofit tests. BUA and SOS: not significant.
Holzapfel et al. [19]	2015	**Disability:** ID **Age:** ACT-19.4 ± 4.9; CV-18.4 ± 3.4; NC-17.0 ± 4.0 **Sample:** 48 - Assisted Cycling Group (ACT): 18 - Voluntary Cycling Group (VC): 16 - Control Group (CON): 14	**Length**: 8 weeks (24 sessions) **Activity**: cycling **Volume**: 30’/session **Frequency**: 3 days/week **Intensity**: - ACT: Throughout the eight weeks, the objective was to increase the assisted pedaling cadence between sessions (from 3 to 5 rpm), but not during the session, up to 60% of the maximum heart rate for their age. - VC: pace is chosen by the subject.	**Body composition and PF:** Chronological age (years), mental age (years), BMI (kg/m^2^), cadence (rpm), power (W), and HR (bpm) **Neuropsychological and psychomotor capacity:** - Purdue Pegboard Test (PPT): unimanual right hand, unimanual left hand, bimanual, RLB, assembly, and combined. - Tower of London (ToL): correct levels, correct moves, time per correct move, and aggregate score	**Significant improvements:** Unimanual dexterity: ACT * (Right: *p* = 0.007; Left: *p* = 0.006) and VC * (Right: *p* = 0.005; Left: *p* = 0.020) NOT in the CON group. Manual dexterity: ATC vs. CON * (*p* = 0.03) VC not significant (*p* 0.11). PPT vs. ToL: correlation of bimanual PPT score and correct movement/time in the ToL (*p* = 0.049). ACT group combined PPT score and correct movement/time in the ToL (*p* = 0.015).
Lee et al. [20]	2016	- **Disability:** ID **Age:** 14–19 **Sample:** 32 - **Control Group (CON):** 15 - **Training Group (TG):** 16	**Length:** 8 weeks **Activity:** balance-focused training **Volume:** 40 min/session **Frequency:** 2 days/week **Intensity:** not specified	**PF:** All subjects were assessed with posture sway and the one-leg stance test (OLS) for postural balance; the timed up-and-go test and 10 m walk test (10 mW) for gait; and the sit-to-stand test (STS) for functional strength.	**TG:** Postural balance *, OLS *, STS *, 10 mW, up-and-go **CON:** Postural balance, OLS, STS, 10 mW, up-and-go **TG vs. CON:** Postural balance *, OLS *, STS, 10 mW, up-and-go
Matute-Llorente et al. [21]	2015	**Disability:** Down Syndrome **Age:** 12–18 **Sample:** 25 - **Control Group (CON):** 14 - **Whole-Body Vibration Group (WBV):** 11	**Length:** 20 weeks **Activity**: WBV: synchronous vibration platform PowerPlate^®^, (Performance Health Systems, LLC, Northbrook, IL). **Volume**: 20’ approx. **Frequency**: 3 days/week **Intensity**: 10 repetitions (30–60 s), 1 min rest, frequency of 25–30 Hz, and peak-to-peak displacement of 2 mm (maximum acceleration of 2.5–3.6 g).	**BMC and BMD** BMC and BMD by DXA and the secondary were bone structure variables by peripheral quantitative computed tomography (pQCT). BMC, BMD, WBTOT (total whole-body), SUBTOT subtotal body (total body less head), ULIMBS (upper limbs), PELV (pelvis), LLIMBS (lower limbs), SPINE (lumbar spine), FNECK (femoral neck), and HIP (total hip).	**WBV vs. CON** BMC (*) BMD (†) (Red: WBV and Blue: CON) - WBTOT *; SUBTOT *†; SPINE *†; FNECK; HIP * **pQCT measurements:** After 20 weeks of training, no significant differences were found between groups. WBV: Tibia: BMD and BMC; Radius: BMD and cortical tissue thickness *. CON: Radius: BMD and BMC.
Matute-Llorente et al. [22]	2015	**Disability:** Down Syndrome **Age:** 12–18 **Sample:** 26 - **Control Group (without DS):** 13 - **DS Group:** 13	**Length:** 20 weeks **Activity:** WBV: synchronous vibration platform (PowerPlate^®^). **Volume:** 20’ approx. **Frequency:** 3 days/week **Intensity:** 10 repetitions (30–60 s), 1 min rest, frequency of 25–30 Hz, and peak-to-peak displacement of 2 mm (maximum acceleration of 2.5–3.6 g).	**BMC and BMD** BMC and BMD by DXA and the secondary were bone structure variables by peripheral quantitative computed tomography.	**SD** CMO * DMO † - WBTOT *, SUBTOT *†, ULIMBS *, PELV *†, LLIMBS *†, SPINE *†, FNECK, HIP† **NO SD** CMO * DMO † - WBTOT *†, SUBTOT *†, ULIMBS *†, PELV *†, LLIMBS *†, SPINE *†, FNECK *†, HIP *† **SD vs. NO-SD (favorable to NO SD)** CMO * DMO † - WBTOT *†, SUBTOT *†, ULIMBS *†, PELV *†, LLIMBS *, SPINE *, FNECK *, HIP
Nackz et al. [23]	2021	**Disability: Down syndrome** **Age:** (CON: 14.4 ± 1.97 years); (TG: 14.9 ± 2.35 years) **Sample:** 22 adolescents (14 boys and 8 girls). - **Control Group (CON):** 11 - **Training Group (TG):** 11	**Length:** 33 weeks **Activity:** swimming **Volume:** 90 min **Frequency:** 3 days/week (M-W-F) **Intensity:** four progressive stages	**Body composition:** bioelectrical impedance device (Tanita MC-980 MA) BMI, FAT (%), FAT (kg), body mass (kg), and height (m) **PF:** Eurofit Test. - Speed of Limb (reps), Handgrip (kg), Balance (N° of Contacts), Flexibility (cm), Sit-Ups (reps), Arm Strength-Endurance (s). Aerobic Capacity. Peak oxygen uptake-VO_2_ peak. - VO_2_ max (L/min), VO_2_ max (mL/kg/min), HR max. Water orientation Test Alyn 3 (WOTA2)	**Body Composition:** BMI *, FAT (%) *, FAT (kg) *, Body Mass (kg) *, Height (m) * **PF:** Speed of Limb (reps), Handgrip (kg) *, Balance (No of Contacts), Flexibility (cm), Sit-Ups (reps) *, Arms Strength-Endurance (s) * **Aerobic Capacity:** VO_2_ max (L/min) *, VO_2_ max (mL/kg/min) *, HR max. **Water orientation Test Alyn 3 (WOTA2)**
Ptomey et al. [24]	2022	**Disability:** ID and developmental with overweight or obesity. **Age:** ~16 years **Sample: 110** - **FTF/CD:** 36 (conventional face-to-face [FTF], conventional meal-plan diet [CD]) - **RD/CD:** 39 (remote delivery [RD]) - **RD/eSLD:** 35 (enhanced traffic light diet [eSLD])	Participants were ASKED TO: **Length:** 6 months **Activity:** any **Volume:** 60 min **Frequency:** 5 or more days/week **Intensity:** moderate to vigorous	**PA level:** Mean physical activity, light activity, and inactivity time were assessed for 7 days at the start of the study and 6 months using a portable accelerometer (ActiGraph wGT3x-BT).	**There was no significant increase in mean PA.**
Ptomey et al. [15]	2021	**Disability:** ID and developmental with overweight or obesity. **Age:** ~16 years **Sample: 110** - **FTF/CD:** 36 - **RD/CD:** 39 - **RD/eSLD:** 35	Participants were ASKED TO: **Length:** 6 months Activity: any **Volume:** 60 min **Frequency:** 5 or more days/week **Intensity:** moderate to vigorous. Progression from 15’/day 3 days/week at week 1 (or current activity level if higher) to 60′/day 5 days/week at week 12 and remaining at that level through 6 months. Participants were also asked to increase their daily steps by 10% each week from their current level until reaching a goal of 10,000 steps per day.	**Body composition:** - Weight - Height - BMI - Waist circumference	**At six months:** **FTF/CD vs. RD/CD:** - Weight - Height - BMI - Waist circumference **RD/CD vs. RD/eSLD:** - Weight * (greater weight loss in DR/eSLD) - Height - BMI * (lower BMI in DR/eSLD) - Waist circumference
Schields et al. [25]	2013	**Disability:** Down’s Syndrome **Age:** 17.9 ± 2.6 **Sample:** 68 - **Social Group (SG):** 34 - **Strength Training Group (STG):** 34	- **Length:** 10 weeks **Strength:** - **Activity:** resistance training (pin-loaded weight machine) - **Frequency**: 2 days/week **Social:** - **Activity:** social activities - **Volume:** 90 min - **Frequency:** 1 day/week	**PA levels and PF:** Work performance, muscle strength, and physical activity levels were assessed at weeks 0, 11, and 24 by an assessor blind to group allocation. - Box stacking test - Weighted pail carry - Chest press 1 RM - Leg press 1 RM - Physical activity	**Significant differences in favor of the ENT group:** **ENT vs. SOC (week 11)** Box stacking test, Weighted pail carry, Chest press 1 RM *, Leg press 1 RM *, PA. **ENT vs. SOC (week 24)** Box stacking test, Weighted pail carry, Chest press 1 RM, Leg press 1 RM *, PA *.
Suárez-Villadat et al. [26]	2023	**Disability:** Down’s Syndrome **Age:** 14.19 ± 2.06 **Sample:** 49 - **Exergame Group (EXE):** 24 - **Control Group (CG):** 25	**Length:** 20 weeks - **Activity:** EXE: Wii-based sports games. Included warm-up, core exercises, and cool-down. CG: Standard PA program focused on motor skills and coordination - **Volume:** 60 min - **Frequency:** 3 day/week	**Body composition:** - BMI - Waist circumference - Hip circumference - Skinfold sum - Body fat (%) - Body adiposity index (%) **PF:** - Handgrip strength (kg) - Standing long jump (cm) - Deep trunk flexibility test (cm) - 10 timed stand tests (s) - 30 s sit-ups (number) - Timed up-and-go test (s) - 6 min walk test (m)	**Significant differences in favor of the EXE group:** Handgrip, 10 timed stand tests (s), 30 s sit-ups (number), timed up-and-go test (s), 6 min walk test (m) Waist circumference, hip circumference, skinfold sum, body fat (%), and body adiposity index (%) **The CG showed no significant differences** in body composition or fitness. **EXE group significant differences:** All PF variables except deep trunk flexibility test (cm). All body composition variables except BMI.
Suárez-Villadat et al. [27]	2024	**Disability:** Down’s Syndrome **Age:** 18.33 ± 1.42 **Sample**: 50 - **Aerobic Training Group (ATG):** 25 - **Strength Training Group (STG):** 25	**Length:** 16 weeks - **Activity:** Strength training (exercises included upper and lower body workouts with progressive weight increases.) vs. aerobic training (aerobic activities focused on endurance and motor development) - **Volume:** 60 min - **Frequency:** 3 day/week	**Body composition:** - BMI - Waist circumference - Hip circumference - Waist-to-height ratio **PF:** - Handgrip strength (kg) - Standing long jump (cm) - Motor fitness (s) - Cardiorespiratory fitness (laps) - 6 min walk test (m)	**The strength group** showed significant improvements in **health-related fitness (*p* < 0.05)** (male group in all variables) but no changes in body composition. **The aerobic program showed no significant changes** in fitness levels or body composition. **Significant differences in favor of the strength group:** Handgrip strength; standing long jump; motor fitness; cardiorespiratory fitness; 6 min walk test.
Suárez-Villadat et al. [28]	2024	- **Disability:** Down’s Syndrome **Age:** 15.5 ± 1.53 years **Sample:** 45 - **Swim Group (SWP):** 15 - **Strength Training Group (STP):** 15 - **Combined Program (CP):** 15	**Length:** 16 weeks - **Activity:** (5 min warm-up, 50 min training, 5 min cool-down). Used front crawl and breaststroke techniques. Strength program: Strength training with free weights targeting upper and lower body Combined (swim + strength): Odd weeks included 1 swimming and 2 strength sessions; even weeks included 2 swimming and 1 strength session - **Volume:** 60 min - **Frequency:** 3 day/week	**Body composition:** - BMI - Waist circumference - Skinfold sum - Body fat (%) **PF:** - Handgrip strength (kg) - Standing long jump (cm) - Deep trunk flexibility test (cm) - 10 timed stand tests (s) - Muscle strength: 1 RM seated bench press and seated leg press. - Timed up-and-go test (s) - 6 min walk test (m)	**Significant differences were observed between SWP and STP** in all variables except for body mass index, thigh skinfold, or deep trunk flexibility test. **Significant differences between CP versus SWP** were shown in hip circumference, triceps skinfold, body fat, handgrip strength (*p* = 0.019), timed up-and-go test, and 30 s sit-ups. **Significant differences between CP versus STP** were observed for waist circumference, hip circumference, triceps skinfold, body fat, chest press 1 RM, leg press 1 RM, 10 timed stand test, and 30 s sit-ups.
Sun et al. [29]	2022	**Disability:** ID **Age:** 12–18 **Sample:** 57 - **Control Group (CON):** 18 - **Training Group (TG)**: 39	**Length**: 9 months **Activity**: moderate-intensity aerobic and resistance exercise **Volume:** 45′/session **Frequency**: 2 days/week **Intensity**: moderate	**PF:** - Aerobic capacity: 9 min run/walk - Grip strength (kg) - Strength–endurance: 30 s sit-ups - Flexibility: sit-and-reach	**TG vs. CON:** - **Aerobic** capacity: 9 min run/walk * (*p* = 0.003) - Grip strength (kg) - Strength–endurance: 30 s sit-ups - Flexibility: sit-and-reach * (*p* = 0.020)
Wang et al. [30]	2022	**Disability:** ID **Age:** 14.17 ± 0.45 **Sample:** 30 - **Control Group (CON):** 15 - **Training Group (TG):** 15	**Length:** 12 weeks (24 sessions) **Activity:** aerobic and resistance exercise **Volume:** 60′. Each session consisted of a 10 min warm-up, a 45 min main exercise (including two 15 min aerobic games, followed by 15 min resistance training), and a 5-minute cool-down. **Frequency:** 2 days/week **Intensity:** All sessions were divided into three levels and each level lasted for four weeks. Exercise intensity increased progressively from 40% to 70% HRR.	**Body Composition:** Weight (kg), BMI, waist circumference, waist-to-height ratio, and %Fat. **PF:** 6-minute walk test, 30 s sit-to-stand, 1 min sit-ups, Hand grip strength (kg), flexibility (sit-and-reach). **Metabolic fitness:** Systolic blood pressure (mmHg), diastolic blood pressure (mmHg)	**Three evaluations: pre-intervention (T1), post-intervention (T2) (12-week protocol), and after 12-week follow-up (T3) (post-protocol + 12 weeks).** **TG vs. CON** **Body composition:** weight (kg) * (T2, T3), BMI * (T2, T3), waist circumference, waist-to-height ratio, and %Fat. **Physical condition:** 6 min walk test * (T2, T3), 30 s sit-to-stand * (T2, T3), 1 min sit-ups, hand grip strength (kg) * (T2), and flexibility (sit-and-reach). **Metabolic fitness:** no significant differences were observed.
Yu et al. [31]	2022	**Disability:** ID **Age:** 12–18 **Sample:** 61 - **Control Group (CON):** 22 - **Training Group (TG):** 40	**Length:** 9 months **Activity:** moderate-intensity aerobic and resistance exercise **Volume:** 45′/session **Frequency:** 2 days/week **Intensity:** moderate	**Body composition:** min-BMI and BMIz - Fat (%) - Weight (kg) - Waist circumference - Waist-to-height ratio	**CON:** - BMI * and BMIz (increase) - Fat (%) - Weight (kg) - Waist circumference - Waist-to-height ratio **TG:** - BMI * and BMIz (decrease) - Fat (%) - Weight (kg) - Waist circumference - Waist-to-height ratio **TG vs. CON:** - BMI * and BMIz - Fat (%) - Weight (kg) - Waist circumference - Waist-to-height ratio
